# ToF-SIMS Imaging for the Analysis of Cholesterol Formation at Macrophage Membrane

**DOI:** 10.3390/metabo15110722

**Published:** 2025-11-05

**Authors:** Mengjiao Sun, Hongzhe Ma, Mingru Liu, Mengchan Xia, Yanhua Chen, Zhaoying Wang, Zhanping Li

**Affiliations:** 1 Key Laboratory of Mass Spectrometry Imaging and Metabolomics, State Ethnic Affairs Commission, Center for Imaging and Systems Biology, College of Life and Environmental Sciences, Minzu University of China, Beijing 100081, China; 24302653@muc.edu.cn (M.S.); 23302569@muc.edu.cn (H.M.); 23302556@muc.edu.cn (M.L.); 201901007@muc.edu.cn (Y.C.); 2 National Narcotics Laboratory Beijing Regional Center, Beijing 100164, China; xiamcsci@126.com; 3 Department of Chemistry, Tsinghua University, Beijing 100084, China

**Keywords:** atherosclerosis, macrophages, cholesterol, time-of-flight secondary ion mass spectrometry imaging, cell metabolism

## Abstract

**Background/Objectives**: Atherosclerosis has its development intricately linked to cholesterol accumulation in the artery wall. Macrophages play a crucial role in early-stage cholesterol aggregation during atherosclerosis. Thus, exploring cholesterol formation in macrophages is of great significance for elucidating the development of atherosclerosis. **Methods**: Time-of-flight secondary ion mass spectrometry (ToF-SIMS) is a powerful technique capable of offering high-spatial-resolution 2D and 3D chemical images, making it an ideal method for studying cholesterol distribution at the single-cell level. In this study, we utilized ToF-SIMS to image the cholesterol distribution in macrophages. By incubating macrophages with acetylated low-density lipoprotein (acLDL), we observed the accumulation of cholesterol on the macrophage membrane. **Results and Conclusions**: The results revealed that acLDL promotes cholesterol formation in macrophages, further clarifying the functions and roles of acLDL and cholesterol in the development of atherosclerosis. This research provides valuable insights into the underlying mechanisms of atherosclerosis and may be helpful to the development of novel preventive and therapeutic strategies.

## 1. Introduction

Atherosclerosis and its associated complications represent a predominant cause of mortality globally, thereby posing a substantial threat to human health [1,2,3,4]. The pathogenesis of atherosclerosis is primarily driven by intricate interactions between inflammatory cells, including macrophages, dendritic cells, and lipoproteins [5]. These interactions promote the progressive accumulation of lipids and inflammatory cells within the arterial intima, ultimately leading to the formation of atherosclerotic plaques [1,6,7].

Cholesterol is a critical structural component of mammalian cells. As an integral element of the plasma membrane, it influences the properties and functions of membrane proteins [8,9]. Low-density lipoprotein (LDL) serves as the primary transporter of cholesterol in the bloodstream [10]. During the process of atherogenesis, LDL accumulates and undergoes modification within the arterial walls, leading to the formation of aggregated LDL deposits (agLDL) [11]. These deposits are identified and ingested by macrophages [1,12,13,14,15,16,17]. Upon the clearance of agLDL by macrophages, lysosomal synapses are established to facilitate the digestion of agLDL, a mechanism that enhances cholesterol internalization and contributes to the formation of foam cells [11,12,14,18]. Simultaneously, diverse inflammatory processes are initiated, thereby expediting detrimental circulation [16,19,20]. The apoptosis of these foam cells results in atherosclerotic necrosis, ultimately contributing to the development of atherosclerotic plaques [15,16,17,21,22]. Therefore, investigating cholesterol accumulation in macrophages is of considerable importance for understanding the early pathogenesis of atherosclerosis. In experimental settings, chemically modified forms of LDL, such as acetylated LDL (acLDL), are widely used to mimic lipid-loading and foam cell formation processes, as they are more stable and efficiently internalized by macrophages via scavenger receptor A (SR-A) [12]. Thus, acLDL was utilized to induce cholesterol accumulation in macrophages, serving as an established in vitro model for early atherogenic lipid uptake.

Imaging serves as an effective and intuitive method for examining cholesterol distribution at the single-cell level. Presently, fluorescence techniques are the predominant imaging approaches employed for analyzing cholesterol distribution within cells [23,24]. However, these fluorescence methods necessitate the use of tracking compounds, which may alter the inherent behavior of cholesterol due to its status as a small lipid molecule. In contrast, mass spectrometry imaging (MSI) distinguishes itself from these microscopic techniques by offering the advantage of directly detecting cholesterol distribution without requiring any modifications. Matrix-assisted laser desorption/ionization (MALDI) was initially introduced as a mass spectrometry imaging (MSI) technique in 1985 [25]. However, its application is constrained by limitations in low ionization rate of cholesterol and spatial resolution at the single-cell level, primarily due to challenges associated with matrix crystal formation and laser focusing. In contrast, several studies have demonstrated that nanoscale secondary ion mass spectrometry (NanoSIMS) can be utilized to map cholesterol distribution with exceptionally high spatial resolution [26,27]. Despite this advantage, NanoSIMS necessitates expensive metabolic labeling and is restricted to detecting labeled small fragment ions of cholesterol, rather than the intact cholesterol molecule. Time-of-flight secondary ion mass spectrometry (ToF-SIMS) is a commonly used MSI method with high spatial resolution for studying distributions of lipid and has been found to be a very good tool to image cholesterol at the single cell levels [28,29,30,31,32,33].

In this study, we employed acetyl low density lipoprotein (acLDL) to stimulate macrophages, thereby inducing cholesterol production. ToF-SIMS was utilized to analyze the distribution and dynamic formation processes of cholesterol at the macrophage membrane. We obtained both two-dimensional (2D) and three-dimensional (3D) images of cholesterol distribution on the macrophage membrane using ToF-SIMS, providing detailed insights into the dynamic formation of cholesterol over the incubation period with acLDL in individual cells. The results demonstrated the efficacy of ToF-SIMS in characterizing changes in cholesterol at the single-cell surface, thereby offering a comprehensive understanding of the mechanisms underlying cholesterol accumulation in the process of atherosclerosis.

## 2. Experimental

### 2.1. Reagents and Materials

A cell line of RAW 264.7 was acquired from Shanghai Biological Technology Co., Ltd. (Shanghai, China). DMEM and penicillin-streptomycin were purchased from Gibco (Thermo Fisher Scientific, Waltham, MA, USA). Fetal bovine serum was obtained from HyClone (Cytiva, Logan, UT, USA). A dish of 35 mm in diameter was obtained from Corning Co., Ltd. (Corning, NY, USA) and the Si wafers was obtained from KYKY Technology Co., Ltd. (Beijing, China). Acetylated LDL (acLDL) was obtained from Shanghai Yeasen Biological Co., Ltd. (Shanghai, China). β-cyclodextrin (MβCD) was obtained from Tokyo Chemical Industry, Co., Ltd. (Tokyo, Japan). DI water was prepared from a Milli-Q water purification system (Millipore, Billerica, MA, USA) .

### 2.2. Sample Preparation

RAW 264.7 cells were cultured with DMEM supplemented with 10% fetal bovine serum and 1% penicillin-streptomycin. They were seeded in a 35 mm × 35 mm dish, in which a cleaned Si wafer was placed beforehand. Then cells were incubated in a humidified atmosphere containing 5% CO_2_ at 37 °C with or without 50 μg/mL acLDL for 48 h.

For the time scale experiments, the cells were incubated with 200 μg/mL acLDL for 9 h, 20 h, 27 h, 42 h, 48 h. For the MβCD experiment, 100 µL of a 35 mg/mL aqueous solution of MβCD was added into the dish and the cells were further incubated for another 1 h at 37 °C. Next, all the silicon wafers mentioned above were removed from the culture media and were gently rinsed for 30 s with DI water. Until drying at room temperature, the samples were introduced to the vacuum chamber of ToF-SIMS for analysis.

### 2.3. ToF-SIMS

ToF-SIMS analysis was carried out on a ToF-SIMS V instrument (IONTOF GmbH, Münster, Germany). A 30 keV Bi_3_^+^ LMIG was applied as an analysis source. Positive ion spectra were collected at mass resolution-priority mode (mass resolution of > 6000 obtained at *m*/*z* 27) to verify the accurate signals of cholesterol ions. Positive spectra were calibrated using C_3_H_8_N^+^ (58.07), C_5_H_12_N^+^ (86.10), C_5_H_14_N^+^ (104.11), C_2_H_8_NO_3_P^+^ (125.02), C_5_H_13_NO_3_P^+^ (166.06), C_5_H_15_NO_4_P^+^ (184.07), C_8_H_20_NO_6_P^+^ (224.11), C_27_H_45_^+^ (369.35). For 3D imaging analysis, argon cluster ion source (Ar_n_^+^, where n is approximately 1700, with a total energy of 10 keV) was applied as the sputtering source to facilitate the sequential etching of the sample. The GCIB was operated at an initial current of 10 nA, decreasing slightly to 9.5 nA at the end of sputtering. The sputtering crater size was 300 × 300 μm^2^, with a total accumulated ion dose of approximately 1 × 10^13^ ions, corresponding to a dose density of 1.12 × 10^16^ ions/cm^2^ after 167 layers. The sputtering was operated in non-interlaced mode, with 20 frames acquired per imaging cycle and 1 s sputtering time between frames. All images were obtained using a 30 keV Bi_3_^+^ primary ion beam rastered over a 50 × 50 µm^2^ area (256 × 256 pixels, repetition frequency = 10 kHz), corresponding to a pixel size of ~0.195 µm (≈195 nm) per pixel. Charge compensation on the sample surface was achieved using a flood gun delivering low-energy electrons at approximately 20 eV. Statistical analyses of ToF-SIMS data are described in the following section.

### 2.4. Data Processing and Statistical Analysis

Data acquisition and image reconstruction were performed using SurfaceLab 7 software (IONTOF GmbH,Münster, Germany). Ion intensities and region-of-interest (ROI) signals were normalized to the total ion counts (TIC) on a per-pixel basis prior to analysis. For each condition, 5–15 individual macrophages were analyzed across three independent culture replicates. The averaged ion intensities were used for relative quantification. Statistical significance was evaluated by two-tailed unpaired Student’s *t*-test using TIC-normalized values, with *p* ≤ 0.05 considered statistically significant. Error bars in figures represent standard deviations (SD) of analyzed cells.

### 2.5. Image Correction

During 3D ToF-SIMS imaging, sample tilt and surface roughness can cause spatial misalignment between depth layers, compromising the accuracy of spatial interpretation. To correct for these topographical deviations, a graphical user interface tool, *zCorrectorGUI*, developed in MATLAB (MATLAB R2019b, MathWorks,Inc., Natick, MA, USA), was used to perform *z*-axis correction of the depth profiling datasets. The *m*/*z* 184.07 ion signal was selected as a reference to represent the cell surface morphology and guide the alignment of depth slices. The *m*/*z* 369.35 ion signal, corresponding to cholesterol, was used to reconstruct and visualize its spatial distribution. This correction procedure significantly improved the registration accuracy between depth slices and enhanced the overall spatial consistency and visualization quality of the 3D images.

## 3. Results

### 3.1. Membrane Surface Secondary Ion Changes in Macrophages upon acLDL Stimulation

**Figure 1:**
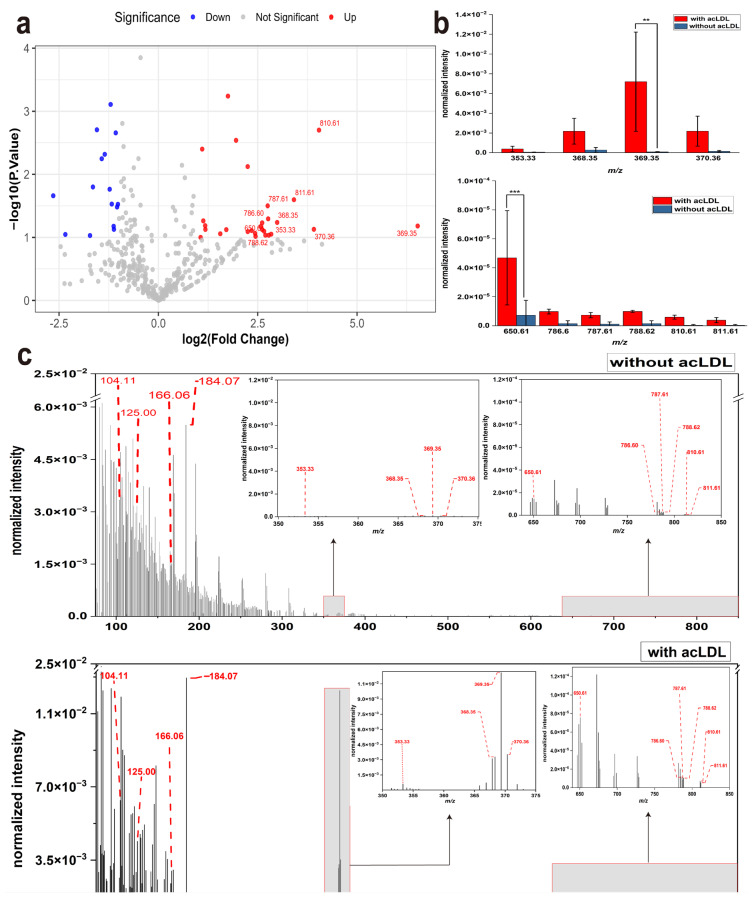
Secondary ion signal profiles of macrophage surfaces with or without acLDL incubation. (**a**) Volcano plot showing the top 10 most significantly altered *m*/*z* features between macrophages incubated with and without acLDL (*p* ≤ 0.1). (**b**) Statistical analysis of these top 10 *m*/*z* values. Significance levels are indicated as follows: ** *p* ≤ 0.01, *** *p* ≤ 0.001. (**c**) Representative ToF-SIMS mass spectra of macrophage membrane surfaces under basal conditions (upper, without acLDL incubation) and after acLDL incubation (lower). The enlarged insets correspond to the *m*/*z* 350–375 and 640–850 ranges, which contain the top 10 most upregulated ions listed in (**a**). Typical phosphocholine-derived fragment ions (*m*/*z* 104.11, 125.02, 166.06, and 184.07) are also indicated as representative markers of membrane phospholipids.

Acetylated low-density lipoprotein (acLDL) is commonly used in atherosclerosis research to simulate early events such as cholesterol accumulation and foam cell formation. It is internalized through scavenger receptors on the macrophage membrane and triggers metabolic remodeling within the cells [12,13,34]. To investigate whether acLDL incubation affects the composition of ion signals on the macrophage membrane surface, this study used RAW 264.7 mouse macrophage cells as a model. The cells were incubated with 50 μg/mL acLDL for 48 h, and then analyzed by ToF-SIMS to examine membrane-surface secondary ion signals in situ after gentle rinsing with deionized water and air-drying at room temperature. The volcano plot was used to represent the changes in the intensities of secondary ion signals (*m*/*z* features) detected on the macrophage membrane surface in the presence of acLDL compared with the incubation without acLDL. To highlight the most significantly altered ion signals, we screened the top ten *m*/*z* peaks that showed statistically significant differences (*p* ≤ 0.1) between the acLDL-treated and control groups. As shown in Figure 1ia, numerous peaks displayed differential abundance when comparing acLDL-treated cells with untreated controls. Some secondary ion signals were upregulated, while others were downregulated. To highlight the most prominent changes, we selected the top 10 *m*/*z* values showing the most significant alterations under the threshold of *p* ≤ 0.1. The top 10 secondary ion signals *m/z* values with the most significant expression changes were 353.33, 368.35, 369.35, 370.36, 650.61, 786.60, 787.61, 788.62, 810.61, and 811.61, all of which were upregulated in expression. Subsequent statistical analysis revealed that *m*/*z* 369.35 was very significantly different, while *m/z* 650.61 showed an extremely significant difference upon acLDL stimulation(Figure 1b). Among the ten most upregulated *m*/*z* values, only *m*/*z* 369.35 and *m*/*z* 650.61 reached very significant and extremely significant levels, respectively. This can be explained by their combination of large fold changes and low intra-group variability. In contrast, other upregulated ions exhibited greater variation among biological replicates or had moderate baseline levels in the control group, which reduced their overall statistical significance despite notable upregulation trends.

To further elucidate the effect of acLDL on the secondary ion signals of the macrophage membrane, these 10 most significantly altered *m/z* peaks were annotated and highlighted in Figure 1c,d. These characteristic ions primarily correspond to cholesterol-related fragments and phosphatidylcholine (PC) species, revealing a dual pattern of lipid remodeling—cholesterol accumulation and phospholipid reorganization. In addition to these lipid species, several characteristic phosphocholine-related fragments at *m*/*z* 104.11, 125.02, 166.06, and 184.07 were also clearly detected, which are typical headgroup ions of PC and serve as representative markers of membrane phospholipids. Among them, *m*/*z* 369.35, was putatively assigned to cholesterol. To validate peak identification, a cholesterol standard was analyzed under identical ToF-SIMS conditions (Supplementary Figure S1), confirming the assignment of *m*/*z* 369.35 to cholesterol. While *m*/*z* 353.33, 368.35 and 370.36 were identified as cholesterol-derived fragments, particularly [M–OH]^+^ and [M–OH+H]^+^ ions. These signals showed marked upregulation following acLDL treatment, suggesting increased cholesterol enrichment on the macrophage membrane surface, a hallmark of early foam cell formation [35,]. The *m*/*z* 650.61 signal is tentatively assigned as a cholesteryl-ester-related secondary ion (e.g., fragments associated with CE 16:0), indicating that cholesterol esterification may occur alongside free-cholesterol accumulation. This further supports an upregulation of esterified cholesterol species following acLDL exposure [29,35]. In addition, *m*/*z* 786.60, 787.61, 788.62, 810.61, and 811.61 were predominantly annotated as PC species with carbon chain lengths ranging from C36 to C38 and multiple degrees of unsaturation. These included PC (38:4), PC (36:2), PC (36:1), and PC (38:6) [36]. These phospholipids are likely enriched in polyunsaturated fatty acids, such as linoleic acid (C18:2) or arachidonic acid (C20:4), based on their typical acyl compositions reported for PC (38:4) and PC (38:6) species in previous studies [36,37]. However, since ToF-SIMS provides only the molecular ion signals without tandem MS fragmentation, the exact fatty acyl chains cannot be directly determined here, and this interpretation should therefore be considered putative rather than definitive [36,37]. Their upregulation in the acLDL-treated group indicates a shift toward a more unsaturated membrane composition, consistent with metabolic remodeling. The annotated identities and biological relevance of these top 10 upregulated *m*/*z* features are summarized in the Supplementary Information Table S1.

Taken together, these results demonstrate that acLDL treatment promotes not only the accumulation and esterification of cholesterol but also the selective enrichment of polyunsaturated phospholipids, suggesting early lipid reprogramming related to foam cell formation and inflammatory activation.

### 3.2. Cholesterol Accumulation on the Macrophage Membrane Induced by acLDL

**Figure 2:**
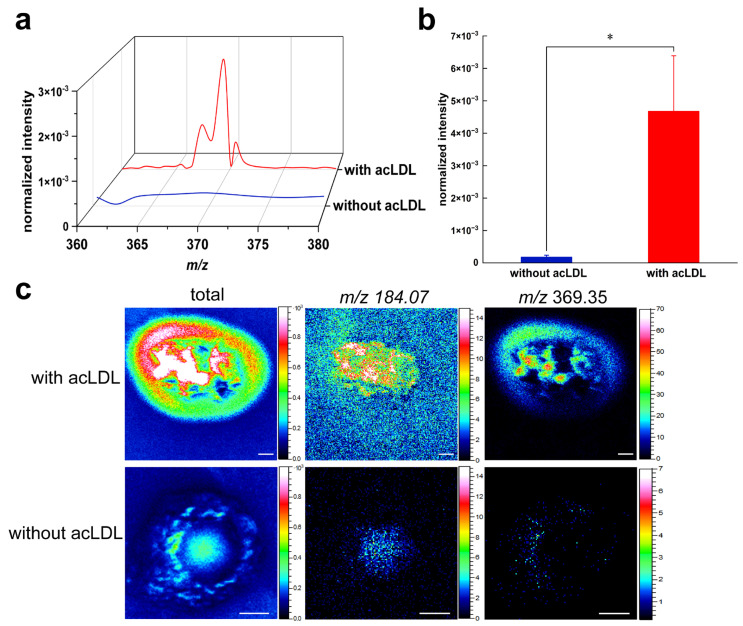
Mass resolution-priority mode analysis of cholesterol secondary ions on macrophage surfaces. (**a**) 3D mass spectrum plot. Overlaid mass-resolution-priority analysis of cholesterol-derived secondary ions detected on macrophage surfaces without acLDL incubation (blue trace) and with acLDL incubation (red trace). (**b**) Relative quantitative comparison of cholesterol secondary ion intensity between macrophages with and without acLDL incubation (* *p* ≤ 0.05; statistical significance indicated if applicable). (**c**) Representative imaging of membrane surface cholesterol distribution in macrophages without and with acLDL incubation, illustrating enhanced cholesterol accumulation upon acLDL incubation. The *m*/*z* 184.07 ion, corresponding to the phosphocholine headgroup of phosphatidylcholine, was included as a reference signal to confirm membrane localization of cholesterol.

In the early stages of atherosclerosis, acLDL can be taken up by macrophages through scavenger receptors, which triggers cholesterol intake, esterification, and foam cell formation [38,39,40]. To investigate acLDL-induced cholesterol formation on the surface of macrophages, we compared mass spectra and ion images obtained with and without acLDL incubation using mass resolution-priority mode (resolving power m/Δm ≈ 6000 at *m*/*z* 27). The peak and bar chart of three-dimensional mass spectrum were analyzed (Figure 2). Figure 2a shows the 3D mass spectrum peaks in the *m*/*z* 350–380 range, comparing the membrane surface secondary ion signals profiles of RAW 264.7 macrophages without acLDL treatment (blue line) with those treated with acLDL (red line). It was shown that at *m*/*z* 369.35, macrophages incubated with acLDL acquired a peak of high intensity, which, as mentioned above, is widely regarded as a cholesterol fragment. However, without acLDL incubation, the signal was weak and could hardly be observed. The significant upregulation of its signal intensity indicates that acLDL induces massive accumulation of free cholesterol on the surface of macrophage membranes. Figure 2b shows a bar graph of relative quantitative analysis of the normalized ion intensity at *m*/*z* 369.35 in the ToF-SIMS data, comparing the acLDL-incubated and unincubated groups. Statistical significance is marked with asterisks (* *p* ≤ 0.05). The results showed that the signal of the treated group was significantly increased (* *p* ≤ 0.05), indicating that a large amount of cholesterol was accumulated on the cell membrane surface. Combined with the 3D peak plots of Figure 2a, this significant difference is not only reflected in the “visual changes”, but also supported by relative quantitative comparison of normalized ion intensities. This trend further supports the key role of acLDL in promoting foam cell formation and demonstrates the reliability of ToF-SIMS for relative quantitative comparison of lipid signals at the single-cell level.

To further visualize the spatial localization of cholesterol ions, representative two-dimensional ToF-SIMS ion images of *m*/*z* 369.35 were obtained for macrophages with and without acLDL incubation. Figure 2c illustrates the two-dimensional spatial distribution images of *m*/*z* 369.35 on the surface of macrophages under conditions without acLDL treatment versus acLDL-treated. As can be seen from the results, the signal at *m*/*z* 369.35 is actually very weak without the incubation of acLDL. Therefore, to aid the observation, the *m*/*z* 184.07 ion image, corresponding to the phosphocholine headgroup of phosphatidylcholine (PC), was used to delineate cell morphology and indicate the position of the cells in the image. This suggests that, in the absence of stimulation, cholesterol distribution is sparse and unconcentrated. However, after incubation with acLDL for a period of time, a large number of cholesterol ions appeared and accumulated on the cell membrane, which could be significantly observed in the 3D mass spectrometry and imaging maps. This suggests that acLDL is actively involved in cholesterol accumulation in macrophages, in line with previous studies as well as the results of the first part of the present study.

These findings provide direct, in situ evidence that acLDL promotes cholesterol accumulation at the macrophage surface. The results also demonstrate the reliability of ToF-SIMS for detecting and comparing lipid distribution at the single-cell level, without requiring fluorescent labeling or chemical modification.

### 3.3. Cholesterol Is Mainly Distributed on the Surface of Macrophage Cell Membrane

**Figure 3:**
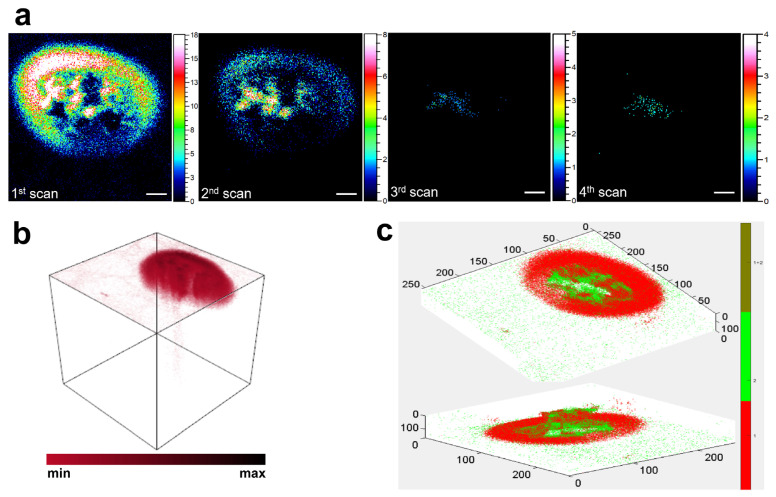
ToF-SIMS images showing the spatial distribution of cholesterol in macrophages. (**a**) Depth profiling images of cholesterol-related ions (*m*/*z* 369.35) acquired using argon cluster ion sputtering. A total of 167 scans were merged into 4 averaged layers to display ion distributions from the cell surface to the interior. Scale bar: 10 μm. (**b**) 3D rendering of cholesterol ion distribution generated from raw ToF-SIMS data using SurfaceLab software (IONTOF). (**c**) *Z*-axis correction map generated using the *zCorrectorGUI* tool in MATLAB. The green channel corresponds to *m*/*z* 184.07 (phosphocholine headgroup of phosphatidylcholine), and the red channel corresponds to *m*/*z* 369.35. The overlay displays the spatial relationship between the two ion signals.

To obtain high-resolution insights into the spatial localization of cholesterol within single macrophages, ToF-SIMS depth profiling was performed using an Ar_n_^+^ gas cluster ion beam, followed by 3D image reconstruction and *z*-axis correction. As shown in Figure 3a, the original 167 depth scans were averaged into four representative layers to reduce noise and improve visualization. The ion signal of *m*/*z* 369.35, putatively assigned to cholesterol, was most intense at the first layer (surface level) and rapidly decreased with increasing sputtering depth. By the third and fourth layers, the signal intensity had nearly diminished to background levels, indicating that the majority of cholesterol ions were confined within approximately the upper 5–10 nm of the cell surface. To further illustrate this spatial distribution, a volumetric rendering of cholesterol ion signals was generated using the SurfaceLab software (Figure 3b). The 3D reconstruction revealed a clear peripheral pattern, with cholesterol ions primarily distributed along the outer edge of the cell volume and minimal signal detected within the intracellular region. This membrane-localized distribution is consistent with the known structural roles of cholesterol in maintaining membrane integrity, regulating lipid rafts, and modulating the function of membrane-bound proteins [29]. To correct for potential sample tilt and ensure accurate spatial alignment along the *z*-axis, we applied the *zCorrectorGUI* tool in MATLAB (Figure 3c). Specifically, the color overlay module within zCorrectorGUI was used to visualize the spatial co-localization of ions. The *m*/*z* 184.07 signal (green channel) was selected to represent cell morphology, while the *m*/*z* 369.35 signal (red channel) was assigned to cholesterol. The resulting color overlay image clearly showed that the cholesterol signal was confined to the outer membrane region, with minimal penetration into the cell interior. This spatial relationship supports the conclusion that cholesterol induced by acLDL exposure accumulates predominantly at the cell surface.

Together, these data confirm that cholesterol induced by acLDL exposure accumulates primarily at the cell membrane surface.

### 3.4. Time-Dependent Accumulation of Cholesterol at the Macrophage Membrane

**Figure 4:**
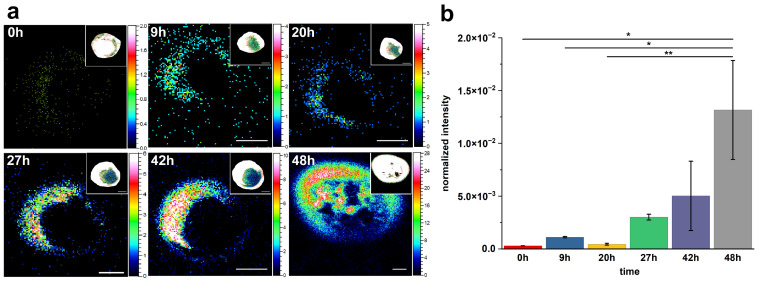
Time-dependent changes in cholesterol distribution on macrophage surfaces following acLDL treatment. (**a**) Representative ToF-SIMS ion images (*m*/*z* 369.35) showing the spatial distribution of cholesterol on RAW 264.7 macrophage surfaces after incubation with 200 μg/mL acLDL for 0 h, 9 h, 20 h, 27 h, 42 h, and 48 h. Insets in the upper right corners show total ion images indicating the positions of macrophage cells. All images were acquired under identical conditions. Scale bar: 10 μm. (**b**) Relative quantitative comparison of cholesterol ion intensity (*m*/*z* 369.35) at each time point. Data represent mean ± s.d. from three independent experiments. Statistical significance: * *p* ≤ 0.05; ** *p* ≤ 0.01 (Student’s *t*-test).

To investigate the temporal dynamics of cholesterol formation on the macrophage surface, ToF-SIMS imaging was performed on RAW 264.7 cells incubated with 200 μg/mL acLDL for varying durations (0 h, 9 h, 20 h, 27 h, 42 h, and 48 h). The secondary ion signal of *m*/*z* 369.35, corresponding to protonated and dehydrated cholesterol ([M+H–H_2_O]^+^), was monitored to track spatial and relative intensity changes. As shown in Figure 4a, minimal cholesterol signal was detected at 0 h, indicating a low basal level of membrane-associated cholesterol under unstimulated conditions. After 9 h of incubation, discrete cholesterol-rich domains began to appear. With further extension of incubation to 20 h and beyond, both the signal intensity and the apparent area of these cholesterol-enriched regions progressively increased, displaying a clear time-dependent accumulation pattern. Statistical comparison of normalized ion intensities (Figure 4b) confirmed this trend. The cholesterol signal remained low at baseline (0 h), but showed a statistically significant increase by 9 h (* *p* ≤ 0.05), followed by a consistent rise through 20 h, 27 h, and 42 h, reaching its peak at 48 h (** *p* ≤ 0.01). Interestingly, despite the large visual difference between 0 h and 48 h, the statistical significance was *p* ≤ 0.05 due to the high intra-group variability at 48 h. In contrast, the comparison between 20 h and 48 h yielded a higher significance level, as the 20 h group exhibited smaller standard deviation. This emphasizes that statistical significance is not solely determined by mean differences, but also by the consistency within biological replicates.

To examine whether the accumulated cholesterol could be removed post-acLDL exposure, we applied a cholesterol depletion strategy using methyl-β-cyclodextrin (MβCD), a well-established agent for extracting free cholesterol from the plasma membrane. However, as shown in Supplementary Figure S2, a 1 h treatment with MβCD at 37 °C failed to significantly reduce cholesterol levels in the acLDL-treated cells. This suggests that the cholesterol was no longer freely extractable, possibly due to integration into membrane domains or conversion into esterified forms.

Collectively, these results demonstrate that acLDL exposure induces a progressive and time-dependent accumulation of cholesterol on the macrophage membrane surface, which becomes resistant to extraction by MβCD. The observed persistence of cholesterol following MβCD treatment indicates that a substantial portion of the accumulated cholesterol remains membrane-associated and cannot be freely removed under standard depletion conditions. Together, the ToF-SIMS and depletion analyses confirm that prolonged acLDL stimulation leads to stable retention of cholesterol at the macrophage plasma membrane.

## 4. Discussion

The present study employed time-of-flight secondary ion mass spectrometry (ToF-SIMS) to visualize and comparatively quantify cholesterol accumulation on macrophage membranes following acLDL stimulation. This approach provided direct, label-free, and high-spatial-resolution insight into the lipid remodeling events associated with early foam cell formation. Our findings collectively demonstrate that acLDL treatment leads to (1) significant changes in the secondary ion signals of the macrophage membrane, particularly enrichment of cholesterol-related and phosphatidylcholine (PC) species; (2) prominent surface localization of cholesterol; and (3) time-dependent, stable retention of cholesterol that becomes resistant to depletion by methyl-β-cyclodextrin (MβCD).

At the metabolic level, acLDL incubation caused a coordinated upregulation of cholesterol fragments and polyunsaturated PC species, as shown by volcano plot and ion annotation analysis. The most strongly upregulated ions (*m*/*z* 369.35, 370.36, and 650.61) correspond to free cholesterol, hydrogenated cholesterol, and cholesteryl ester fragments, respectively, highlighting both accumulation and esterification processes. Meanwhile, the increased abundance of polyunsaturated PC species such as PC (38:4), PC (36:2), and PC (38:6) indicates a shift toward a more unsaturated and dynamic membrane environment. These compositional changes imply that acLDL stimulation not only triggers cholesterol deposition but also drives structural reorganization of the lipid bilayer to accommodate altered lipid loading. Such lipid remodeling is consistent with previous reports describing early membrane reprogramming in macrophage-derived foam cells [29,35,,36].

Spatially resolved ToF-SIMS imaging revealed that cholesterol accumulation induced by acLDL occurred predominantly at the plasma membrane surface, as supported by both two-dimensional and three-dimensional imaging. Depth profiling confirmed that the cholesterol-associated ion (*m*/*z* 369.35) signal was mainly confined within the top 5–10 nm of the cell, with minimal penetration into the intracellular region. This surface-localized distribution supports the notion that cholesterol first integrates into the membrane before further intracellular trafficking or esterification. The localization pattern also aligns with the physiological role of the plasma membrane as a reservoir and regulatory interface for cholesterol homeostasis, maintaining lipid raft integrity and signal transduction balance [28,41,42].

The time-course analysis further revealed a gradual and significant increase in cholesterol signal intensity from 0 h to 48 h after acLDL exposure, consistent with progressive accumulation at the membrane surface. Importantly, when cholesterol-depleted conditions were applied using MβCD, no substantial decrease in cholesterol signal was observed, indicating that the accumulated cholesterol was not freely extractable. This resistance to MβCD-mediated depletion suggests that prolonged acLDL stimulation leads to structural stabilization of cholesterol within the membrane domain or partial conversion into esterified forms. Together, these results confirm that once macrophages undergo acLDL-induced lipid remodeling, membrane-bound cholesterol becomes structurally embedded and less responsive to acute depletion treatments.

From a methodological standpoint, ToF-SIMS provides a unique advantage over traditional biochemical or fluorescence-based assays by enabling direct, label-free, and high-resolution mapping of endogenous lipids under native cellular conditions. This technique captures both spatial and compositional information at the single-cell level, making it particularly powerful for studying lipid microdomain formation, cholesterol dynamics, and foam cell differentiation. By integrating relative ion analysis, depth profiling, and time-course imaging, ToF-SIMS offers a comprehensive platform for investigating early lipid dysregulation events underlying atherosclerotic initiation.

Although acetylated LDL (acLDL) differs chemically from the aggregated LDL (agLDL) that accumulates in atherosclerotic lesions in vivo, it serves as a reproducible and well-established in vitro model to study scavenger receptor–mediated lipid uptake and foam cell formation in macrophages.

## 5. Conclusions

In summary, this study establishes a spatiotemporal framework of cholesterol accumulation during acLDL-induced macrophage activation. The results reveal that cholesterol deposition begins at the membrane surface, increases in a time-dependent manner, and becomes resistant to extraction under sustained acLDL exposure. These findings not only highlight the pivotal role of cholesterol homeostasis in foam cell formation but also demonstrate the capacity of ToF-SIMS as a precise and informative tool for probing membrane lipid remodeling in situ.

## Data Availability

The original contributions presented in this study are included in the article/Supplementary Materials. Further inquiries can be directed to the corresponding authors.

## Supplementary Materials

The following supporting information can be downloaded at: https://www.mdpi.com/article/10.3390/metabo15110722/s1. Table S1: Tentatively assigned secondary ion species detected on macrophage membranes by ToF-SIMS analysis; Figure S1: Validation of cholesterol ion assignment by comparison with a cholesterol standard; Figure S2: Effect of methyl-β-cyclodextrin (MβCD) on cholesterol accumulation at the macrophage surface.
