# Supplementary material for: ToF-SIMS Imaging for the Analysis of Cholesterol Formation at Macrophage Membrane
Source: Metabolites. 2025 Nov 5;15(11):722. doi: 10.3390/metabo15110722 (PMC12654415; doi:10.3390/metabo15110722)
Supplement: Supplementary file 1 [file metabolites-15-00722-s001.zip › metabolites-3929674-supplementary.pdf]

Supplementary Material

1.Supplementary Table

m/z	**Tentatively Assigned Secondary Ion Species**	**Putative Biological Relevance**
104.11	Phosphocholine-related secondary ion ($C_5H_{14}N^+$)	Characteristic headgroup ion of phosphatidylcholine (PC); indicator of membrane phospholipids
125.02	Phosphoethanolamine-related secondary ion ($C_2H_8NO_3P^+$)	Fragment of phospholipid backbone; reflects membrane composition
166.06	Phosphocholine-related secondary ion ($C_5H_{13}NO_3P^+$)	Diagnostic ion of PC and sphingomyelin; marker of membrane integrity
184.07	Phosphocholine headgroup secondary ion ($C_5H_{15}NO_4P^+$)	Major PC headgroup signal; used for cell morphology delineation in ToF-SIMS images
353.33	Cholesterol-related secondary ion ($[M+H–2H_2O]^+$)	Minor fragment associated with cholesterol accumulation
368.35	Cholesterol-related secondary ion $[M−H_2O]^+$	Indicative of cholesterol presence
369.35	Protonated and dehydrated cholesterol-related species ($[M+H–H_2O]^+$)	Primary signal representing cholesterol enrichment
370.36	Cholesterol +1 isotope peak $[M+H−H_2O]^+$ (+1 iso)	Rearranged product suggesting cholesterol enrichment
650.61	Cholesteryl ester-related secondary ion	Indicator of cholesterol esterification
786.60	Phosphatidylcholine-related secondary ion (PC 38:4 or PC 36:2)	May reflect membrane composition/remodeling
787.61	Isotopic peak of PC (36:2)	Enhancement of membrane unsaturation
788.62	Phosphatidylcholine-related secondary ion (PC 36:1)	Membrane structural regulation
810.61	Phosphatidylcholine-related secondary ion (PC 38:6)	May reflect membrane composition/remodeling
811.61	Phosphatidylcholine-related secondary ion (PC 36:4 or PC 38:4)	Lipid signaling and membrane remodeling

Table S1. Tentatively assigned secondary ion species detected on macrophage membranes by ToF-SIMS analysis.

This table summarizes a subset of secondary ion signals (*m/z* values) detected on the macrophage membrane surface, including both (i) the top 10 most upregulated *m/z* ions in macrophage membranes upon acLDL treatment identified from volcano-plot analysis, and (ii) the characteristic phosphocholine-related ions (*m/z* 104.11, 125.02, 166.06, 184.07) used for morphological reference and mass calibration.

Each entry lists the tentative assignment and its putative biological relevance. Because no tandem MS (MS/MS) verification was performed, all assignments are tentative and based on previously reported ToF-SIMS fragment ions of cholesterol and phosphatidylcholine species.

The cholesterol and cholesteryl-ester-related secondary ions reflect lipid accumulation and esterification processes at the macrophage membrane, whereas the phosphocholine-related ions serve as internal references to visualize cell morphology.

2.Supplementary Figure

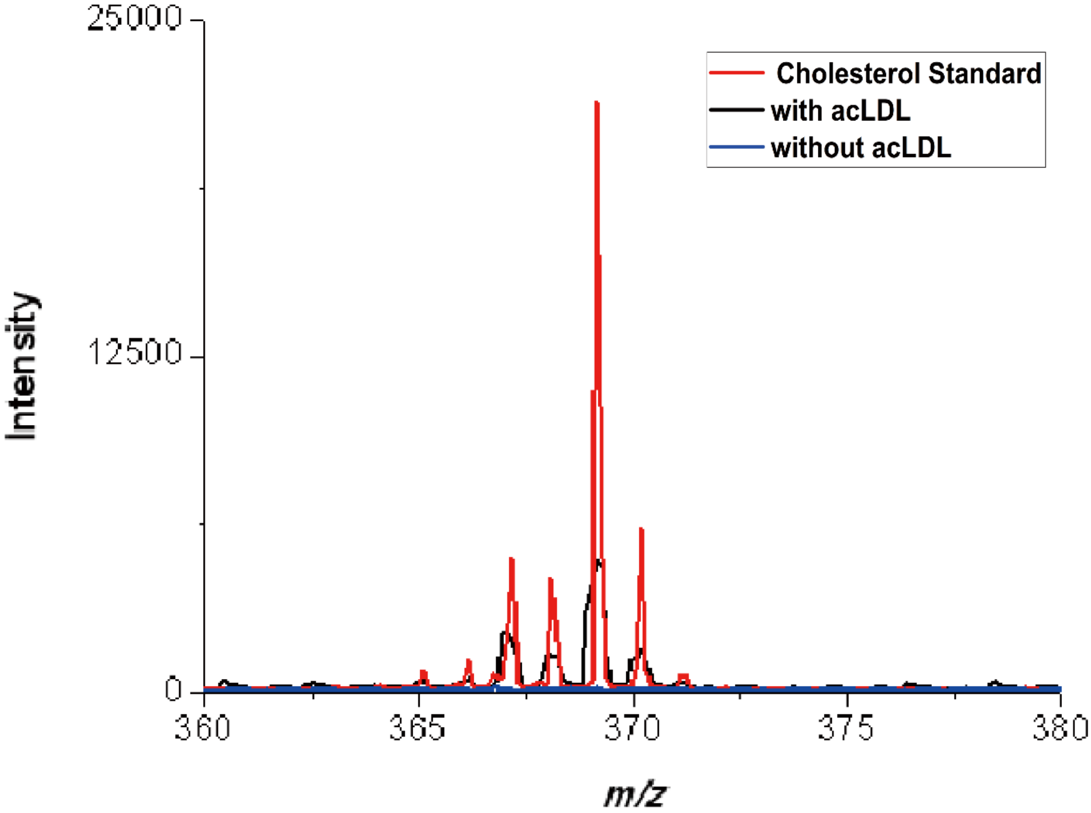

Figure S1. Validation of cholesterol ion assignment by comparison with a cholesterol standard.Overlaid ToF-SIMS spectra in the *m/z* 360–380 range were acquired under identical conditions for a cholesterol standard (red), RAW 264.7 macrophages incubated with acLDL (black), and untreated macrophages (blue). The characteristic cholesterol ion at *m/z* 369.35 ($[M+H–H_2O]^+$) and its adjacent fragments appear prominently in the cholesterol standard and the acLDL-treated cells, but are nearly absent in the untreated group.

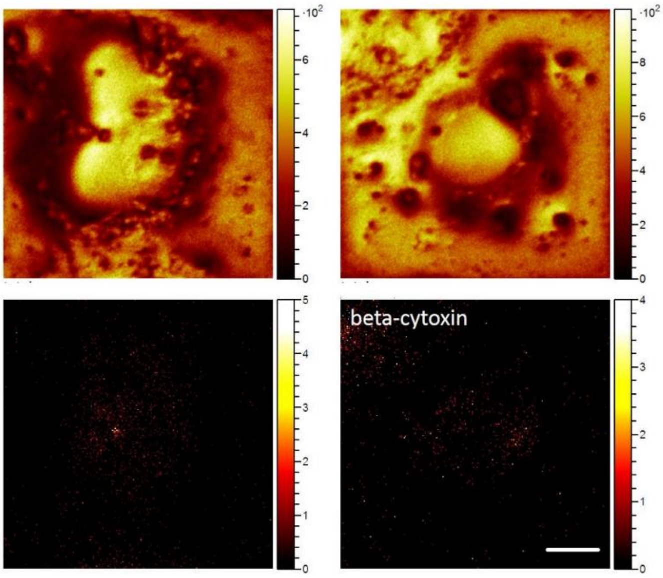

Figure S2. Effect of methyl-β-cyclodextrin (MβCD) on cholesterol accumulation at the macrophage surface.RAW 264.7 cells were treated with 200 µg/mL acLDL for 24 h. To assess the reversibility of cholesterol accumulation, cells were either untreated (A) or incubated with 35 mg/mL MβCD for 1 h at 37 °C (B). ToF-SIMS imaging (*m/z* 369.35) revealed no substantial decrease in cholesterol signal intensity, suggesting that cholesterol deposits formed after acLDL treatment were resistant to MβCD-mediated extraction.
